# Complete Genome Sequence of Acute Hepatopancreatic Necrosis Disease-Causing *Vibrio campbellii* LA16-V1, Isolated from *Penaeus vannamei* Cultured in a Latin American Country

**DOI:** 10.1128/genomeA.01011-17

**Published:** 2017-09-14

**Authors:** Ye Som Ahn, Patharapol Piamsomboon, Kathy F. J. Tang, Jee Eun Han, Ji Hyung Kim

**Affiliations:** a Infectious Disease Research Center, Korea Research Institute of Bioscience and Biotechnology, Daejeon, Republic of Korea; b Department of Biochemistry, Chungnam National University, Daejeon, Republic of Korea; c Faculty of Veterinary Science, Prince of Songkla University, Songkhla, Thailand; d ShrimpVet Laboratory, Ho Chi Minh City, Vietnam; e CJ CheilJedang Feed and Livestock Research Institute, Suwon, Republic of Korea

## Abstract

We report here the complete genome sequence of *Vibrio campbellii*, isolated from *Penaeus vannamei* cultured in a Latin American country. The Tn*3*-like transposon and *pirAB* genes were encoded on the plasmid pLA16-2. These data support the geographical variations in the virulence plasmid found among acute hepatopancreatic necrosis disease (AHPND)-causing *Vibrio* isolates from Latin America and Asia.

## GENOME ANNOUNCEMENT

Acute hepatopancreatic necrosis disease (AHPND), which is caused by *Vibrio* spp. carrying a virulence plasmid encoding the *pirAB* gene, has caused significant economic losses in the shrimp-farming industry (1). Initially, *V. parahaemolyticus* was the only species known to cause this disease; later, other AHPND-causing *Vibrio* spp., such as *V. owensii* and *V. campbellii*, were also reported (2–5). Recent studies have also shown that there are geographical variations in the virulence plasmid among AHPND isolates (6). There is limited information for other AHPND-causing *Vibrio* spp., and so we present here the complete genome of *V. campbellii* LA16-V1, isolated from *Penaeus vannamei* cultured in a Latin American country.

Genomic DNA was isolated using a DNeasy blood and tissue kit (Qiagen). Sequencing was performed by Macrogen, Inc., using a hybrid approach with the PacBio RS II (Pacific Biosciences) (20-kb SMRTbell template library) and Illumina HiSeq 2000 (paired-end short-read data) platforms. The PacBio long-read data (1,787,793,760 bp, 255,476 reads) were *de novo* assembled by FALCON version 0.2.1, and the Illumina paired-end reads (1,053,525,915 bp, 10,439,662 reads) were mapped to the assembled contigs to improve the accuracy of the genome. Annotation was carried out using the NCBI’s Prokaryotic Genome Annotation Pipeline (http://www.ncbi.nlm.nih.gov/books/NBK174280).

The fully assembled genome contained 6,124,368 bp consisting of two chromosomes, designated Chr I (3,579,637 bp) and Chr II (2,230,177 bp), and a total of four plasmids, designated pLA16-1 (109,793 bp), pLA16-2 (73,461 bp), pLA16-3 (67,308 bp), and pLA16-4 (63,992 bp). The two chromosomes showed similar G+C contents (45.6% and 45.4%) and percentages of coding regions (87.1% and 86.8%). Moreover, most of the predicted tRNAs (*n* = 133), rRNA (*n* = 37), and noncoding RNAs (*n* = 4) were encoded on Chr I, except 15 tRNAs and 3 rRNAs on Chr II and one tRNA on pLA16-1. Overall genome similarities among LA16-V1 and other completely sequenced *V. campbellii* strains were assessed using the orthologous average nucleotide identity algorithm (7), and the results indicated 96.7% and 97.9% genome similarities to those of *V. campbellii* ATCC BAA-1116 (8) and *V. campbellii* 20130629003S01 (9), respectively.

The *pirAB*-containing region that comprises the homologue counterparts of the *pirAB* and *pirAB*-Tn*903* composite transposons was encoded on the plasmid pLA16-2. The *pirAB*-containing region was almost identical (>99%) to those from the AHPND-related plasmids pVPE61a (10), pVA1 (11), and pVPA3-1 (1) identified in *V. parahaemolyticus*, plasmid pVHvo (12) in *V. owensii*, and plasmid pVCGX1 (8) in *V. campbellii*. Similar to the previous report (6), the pLA16-2 contained a 3-kb Tn*3*-like transposon that is absent in pVCGX1 in *V. campbellii* 20130629003S01 isolated from China, as well as other plasmids in *V. parahaemolyticus* from southeast Asia, thus supporting the geographical variations in the virulence plasmid found among AHPND-related *Vibrio* isolates from Latin America and Asia. The genome of *V. campbellii* LA16-V1 provides important insights for the study of virulence plasmids to facilitate AHPND control in shrimp aquaculture.

### Accession number(s).

The genome of *V. campbellii* strain LA16-V1 has been deposited at DDBJ/ENA/GenBank under the accession numbers CP021145 (Chr I), CP021146 (Chr II), CP021147 (pLA16-1), CP021148 (pLA16-2), CP021149 (pLA16-3), and CP021150 (pLA16-4).
